# Molecular and Phylogenetic Analysis of Bovine Papillomavirus Type 1: First Report in Iraqi Cattle

**DOI:** 10.1155/2016/2143024

**Published:** 2016-06-20

**Authors:** Mohammed A. Hamad, Ahmed M. Al-Shammari, Shoni M. Odisho, Nahi Y. Yaseen

**Affiliations:** ^1^College of Veterinary Medicine, Al-Fallujah University, Al-Anbar 31002, Iraq; ^2^Iraqi Center for Cancer and Medical Genetic Research, Al-Mustansiriya University, Al-Qadisiyah, Baghdad 1001, Iraq; ^3^College of Veterinary Medicine, Baghdad University, Baghdad 1001, Iraq

## Abstract

This study aimed to provide the first molecular characterization of bovine papillomavirus type 1 (BPV-1) in Iraq. BPV is a widely spread oncogenic virus in Iraqi cattle and is associated with the formation of both benign and malignant lesions, resulting in notable economic losses in dairy and beef cattle. In the current study, 140 cutaneous papilloma specimens were collected from cattle in central Iraq. These samples were submitted to histopathological examination, PCR, and sequencing analysis. The histopathology revealed that the main lesion type among the specimens was fibropapilloma. BPV-1 DNA was detected in 121 of the samples (86.42%) in Iraqi cattle as the main causative agent for the disease. A partial sequence for the E2, L2 genes, and complete sequence for the E5 gene were deposited in GenBank. Phylogenetic analysis confirmed the presence of BPV-1 and showed that the origin of infection may be imported European cattle. Obtaining a complete E5 gene sequence enabled us to perform structural predictions. This study presents the first report of BPV-1 infection in the Iraqi cattle and contributes to extending the knowledge of the origin of the spread of this disease. The results of this study will aid in the development of appropriate control measures and therapeutic strategies.

## 1. Introduction

Bovine papillomavirus (BPV) contains a double-stranded, circular, 8 kb DNA genome. BPVs display tropism for mucosal tissues and squamous epithelium as well as mesenchymal tissue [1] and are associated with the development of both benign and malignant lesions [2, 3]. Although BPVs are species-specific viruses, BPV-1, BPV-2, and BPV-13 may infect equids as well as cattle and can cause the development of tumors in these species [4–6]. Fourteen BPV types have been defined, and they are classified into four genera:* Xipapillomavirus*,* Deltapapillomavirus*,* Epsilonpapillomavirus,* and* Dyoxipapillomavirus* [7–9]. E5, a small membrane-associated protein, is the main transforming protein of BPV and possesses a strong biological effect [10]. The E5 protein induces cellular transformation through its stimulation of platelet-derived growth factor receptor beta (PDGF*β*-R) [11]. Furthermore, reductions in the expression of major histocompatibility complex class I (MHCI) molecules on the cell surface enable the virus to evade immunosurveillance, leading to the inhibition of intracellular transport by means of abnormal connexin expression [5]. Reference [12] documented a tricomponent complex composed of E5/PDGF*β*R/subunit D in vivo. E5 oncoprotein was previously shown to bind to the proteolipidic D component of V1-ATPase proton pump. Reference [12] suggests that the E5/PDGF*β*R/subunit D complex may perturb proteostasis, organelle, and cytosol homeostasis, which can result in altered protein degradation and in autophagic responses. Reference [13] proposed that E2 has a role in the initiation of BPV DNA replication by support E1 binding to the BPV origin via DNA-protein and protein-protein interactions. E2 is a multifunctional protein that serves main roles in transcriptional activation and genome maintenance and cooperates with the viral E1 helicase to initiate viral DNA replication. The BPV genome contains seventeen E2 binding sites, mainly concentrated within the long control region, and a single E1 binding site at the origin of viral replication [14]. Another study by [15] suggests the important participation of L2 protein in the packaging of BPV genome within PV virions, which involves interaction of L2 protein with specific DNA sequences.

In Iraq, our group previously investigated methods for growing BPV in cell cultures derived from skin warts collected from cattle to establish a cell culture technique to enable further studies. Cells from abdominal skin, neck, and udders were cultured, and the cultured cells show both epithelial and mesenchymal morphology. Successful long-term culture was achieved, and the cultured cells were used to prepare vaccines for the treatment of papillomas in BPV-infected cattle [16]. The Holstein and crossbred Holstein Friesian breeds are the main types of cattle in Iraq and are an important source of dairy and beef products [17, 18]. Bovine papillomatosis, caused by BPV genotypes, is responsible for significant economic losses due to the associated growth retardation, weight loss, and decreased milk production in BPV-infected animals [19]. For these reasons, gaining knowledge about this disease is of importance to cattle breeders in Iraq. The natural carrier and primary source of BPV is cattle. The virus enters the body through scratches or other injuries, and infection occurs via both direct and indirect contact. The infection appears to be spread through contact with contaminated materials, milking machines, and semen. Other factors, including malnutrition, hormonal imbalances, mutations, and long-term exposure to sunlight, can increase the risk of infection in cases of immunodeficiency [20]. Moreover, peripheral blood mononuclear cells were shown to be a reservoir of BPV-1 and BPV-2 DNA in affected animals [21].

This study aimed to identify and characterize BPV-1 that is circulating in central Iraq. The results of this study will aid in the development of appropriate control measures and therapeutic strategies.

## 2. Materials and Methods

### 2.1. Animals and Sample Collection

In this study, 140 cutaneous papilloma samples were collected from 140 farm cattle in different areas in central Iraq by registered veterinarians (Anbar, Baghdad, and Diwaniyah cities). The study sample included cows suffering from bovine papillomatosis that were brought to private clinics by their owners from December 2013 to May 2015. Specimens were collected for the study during routine treatment and care as well as through site visits to selected farms. The collected samples had varying diameters (from 1 to 10 cm) and came from different parts of the body (e.g., udder, teat, abdomen, and back). Each sample was immediately divided into 2 parts, which were either frozen in a deep freezer for subsequent molecular biology analysis or fixed in 10% neutral buffered formalin for histological analysis.

### 2.2. Histopathology

Tissue samples were fixed in formalin and processed by standard techniques. The samples were cut into 5 *μ*m thick sections, placed on slides, and stained using hematoxylin and eosin (H&E). The slides were evaluated under a microscope at different magnification.

### 2.3. PCR

A Bosphore® Tissue Genomic Manual DNA Extraction Spin Kit (Anatolia Geneworks, Turkey) and a Magnesia® Genomic DNA Tissue Kit (automated Magnesia DNA Extraction machine) (Anatolia Geneworks, Turkey) were used for this study. The kits were used to extract DNA from 140 frozen tissue samples according to the manufacturer's instructions. To accomplish this, a specifically designed primer set for BPV-1 was used (forward 5′-AGGAGGGTCATGCTTTGCTC-3′; reverse 5′-GCTGTTCGGAGTGGTGTGTA-3′) to obtain DNA fragments of 847 bp. These primers were designed to target conserved regions (identical nucleic acid sequence) for alignment of the following BPV type 1 complete genomes (X02346, NC_001522, AB626705, and JX678969). This newly designed primer was validated including testing for inclusivity and exclusivity. Amplification was performed using a SureCycler 8800 Thermal Cycler (Agilent Technologies, USA) in a final volume of 25 *μ*L, containing 100–300 ng DNA, 2 mM MgCl_2_, 1.25 *μ*L primers (0.5 *μ*M), and 12.5 *μ*L 1X KAPA2G Robust HotStart ReadyMix (Kapa Biosystems, Cape Town, South Africa). The amplification protocol used for this work is shown in Table 1. The PCR products of the viral DNA were detected by electrophoresis on a 1.5% agarose gel containing ethidium bromide, which was placed in TBE buffer and run at a constant voltage (100 V) for approximately 35 min. DNA was visualized using a VISION Gel Documentation System (Scie-Plas, UK). As a negative control, DNA was extracted from skin tissues collected from clinically healthy slaughtered cattle that had previously shown negative results by both histological and PCR analysis.

### 2.4. Sequencing

A total of 121 BPV-1-positive specimens (as determined by PCR), which were selected as representative of BPV's geographical distribution, were sequenced to confirm viral genome type. For sequencing analysis, PCR products which contain E2, E5, and L2 genes were purified using a StrataPrep DNA Gel Extraction Kit (Agilent Technologies, USA); sequencing was performed at the National Instrumentation Center for Environmental Management (NICEM), College of Agriculture and Life Sciences, Seoul National University (South Korea). Sequencing reactions were performed using both forward and reverse primers in a 10 *μ*L total reaction volume (ABI BigDYE V3.1 Ready-Reaction Kit; Applied Biosystems, USA) according to the manufacturer's recommendations. The samples were analyzed on a 3730XL DNA Analyzer (Applied Biosystems, USA). Forward and reverse complementary sequences were aligned using ApE (A plasmid Editor) software (v2.0.49, 2015), and the obtained results were submitted to GenBank and analyzed via BLAST search (http://blast.ncbi.nlm.nih.gov/) on the GenBank database. ApE software was used to detect corresponding amino acid sequences.

### 2.5. Phylogenetic Analysis

The sequences obtained in this study were submitted to phylogenetic analysis. As described above, the sequences were generated using a BPV-1-specific primer set, and the products were deposited in GenBank under accession number KT203919, along with the following reference strains for all 13 BPV genotypes: BPV-1 (accession number X02346), BPV-2 (accession number M20219), BPV-3 (accession number NC_004197), BPV-4 (accession number X05817), BPV-5 (accession number AJ620206), BPV-6 (accession number AJ620208), BPV-7 (accession number DQ217793), BPV-8 (accession number DQ098913), BPV-9 (accession number AB331650), BPV-10 (accession number AB331651), BPV-11 (accession number AB543507), BPV-12 (accession number JF834523), and BPV-13 (accession number JQ798171). Sequences were aligned using ApE software. To identify evolutionary relationships among the analyzed sequences, phylogenetic analysis was performed via the neighbor-joining method using MEGA software version 6 [22].

### 2.6. Protein Structure

To study the protein structures corresponding to the sequenced genes, we used the I-TASSER server, which is an Internet-based software product that enables protein structure and function predictions. I-TASSER allows automated generation of high-quality predictions of the 3D structures and biological functions of protein molecules based on their amino acid sequences [23, 24].

## 3. Results

### 3.1. Histopathology

**Figure 1:**
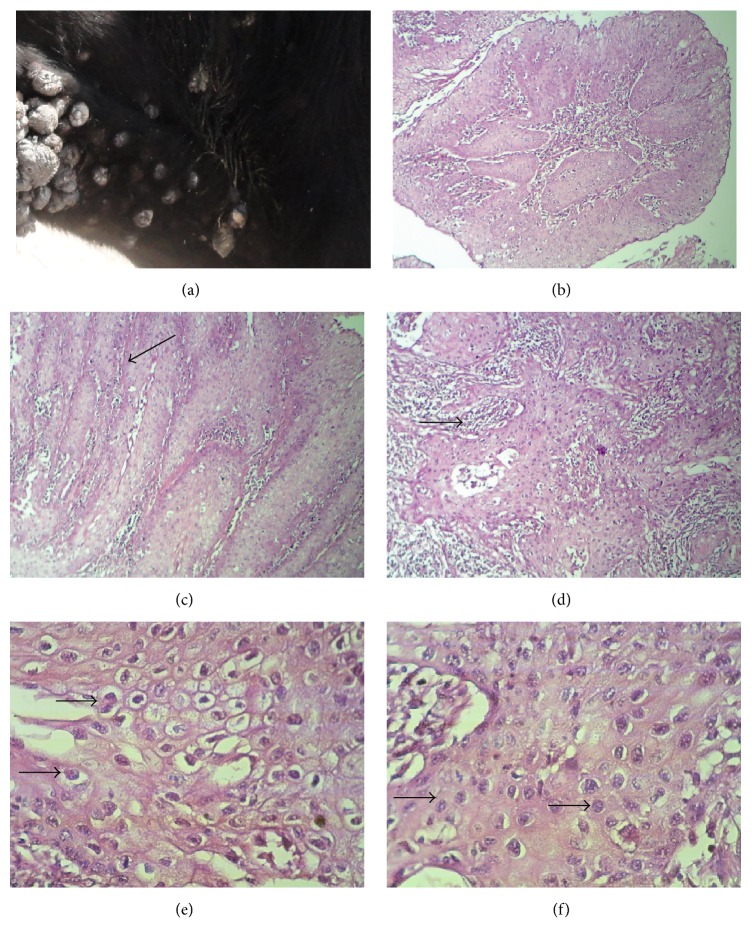
Cutaneous papillomas of the skin. (a) Neck, cow. Multiple exophytic, irregular verrucous papilloma masses arising in the skin. (b) Histopathological section showing papillary projections composed of a hyperkeratotic epidermis with a central collagenous core (10x). (c) Interdigitated epidermal and dermal papillary projections (shown by arrows, 10x). (d) Fibroblast proliferation, collagen deposition, and lymphocyte infiltration (shown by arrows, 10x). (e) Koilocytes (arrows) and keratinocytes with swollen perinuclear halos (40x). (f) Koilocytes with clear cytoplasm and pyknotic nuclei (shown by arrows, 40x, stained with H&E).

All 140 of the collected samples could be clinically described by the presence of multiple exophytic warts (Figure 1(a)). Most of them located on the head and neck (46.6%), udder and teats (19%), legs (16%), and back and abdomen (18.4%). Histopathological examination indicated that the samples were fibropapilloma. In Figures 1(b) and 1(c), the epidermal and dermal interdigitations (papillary projections) of representative samples are shown. Fibroblast proliferation, collagen deposition, and lymphocyte infiltration were observed (Figure 1(d)). Furthermore, koilocytes that are keratinocytes with perinuclear halos or with swollen, clear cytoplasm and pyknotic nuclei were present (Figures 1(e) and 1(f)).

### 3.2. PCR

**Figure 2:**
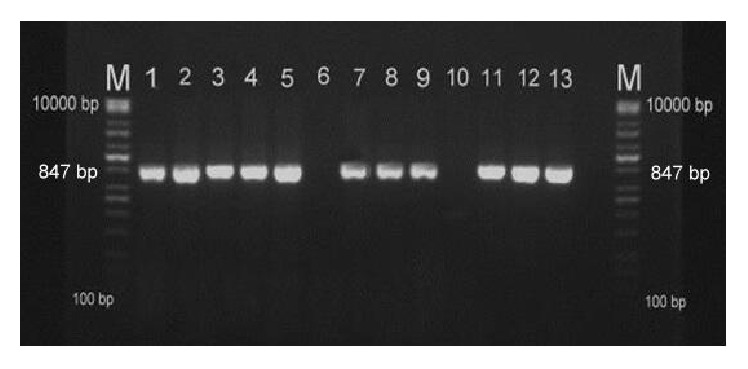
Skin wart samples. PCR products for E2, E5, and L2 genes visualized in an ethidium bromide-stained 1.5% agarose gel following electrophoresis in TBE buffer. M: 100–10,000 bp marker; lanes: 1–5, 7–9, and 11–13: BPV-1-positive samples with bands at 847 bp; lane 6: no sample; lane 10: negative control.

BPV-1 DNA was detected in 121 of the 140 collected papilloma samples. These BPV-1-positive samples produced DNA fragments of 847 bp in length for E2, E5, and L2 genes, which were amplified using BPV-1-specific primers (Figure 2). PCR analysis showed that 86.42% of the analyzed bovine papillomatoses were induced by BPV-1 in the assessed Iraqi cattle populations, which included both imported breeds and crossbreeds being raised in central Iraq.

### 3.3. Sequencing

Sequencing analysis was conducted on the PCR products for E2, E5, and L2 genes amplified from the collected samples that showed identical sequence. The results confirmed the presence of BPV-1, with 97% identity for the majority of the NCBI BLAST-searched BPV-1 sequences. As we used BPV-1-specific primers to amplify the extracted DNA samples, BPV-1 was the predominant genotype detected in all 121 BPV-positive (100%) cattle wart tissue samples. This finding is the first genotyped confirmation of the presence of BPV-1 as a primary causative agent for bovine papillomatosis in Iraqi cattle in the central Iraqi region.

### 3.4. Phylogenetic Analysis

**Figure 3:**
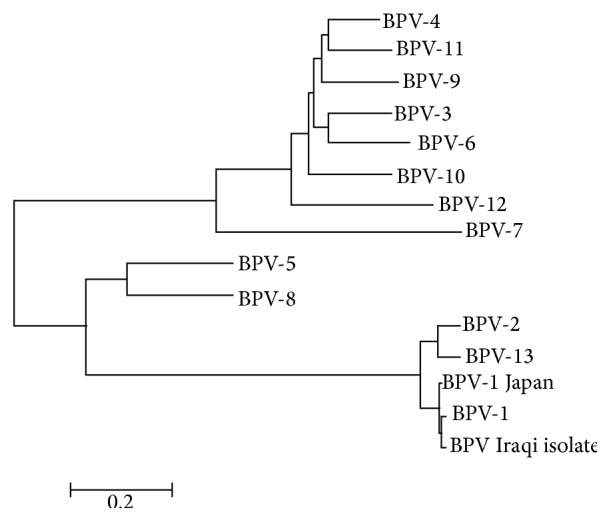
Phylogenetic tree showing the identified Iraqi BPV sequence. This is the first report of a BPV-1 sequence in Iraq (KT203919); the sequence was found to belong to the genus* Deltapapillomavirus*. The sequence was constructed via the neighbor-joining method using MEGA 6 software.

A phylogenetic tree was generated using retrieved genome sequences that were deposited under accession number KT203919 and analyzed against 13 BPV genotype reference sequences (Figure 3). BPV-1 was the main BPV genotype identified (121 samples). The aligned sample sequences were classified as BPV-1 (genus* Deltapapillomavirus*). The aligned sequences showed high similarity both to the nucleotide sequence of BPV-1 (accession number X02346) and to a sequence of a BPV isolate from Japan (accession number AB626705).

**Figure 4:**
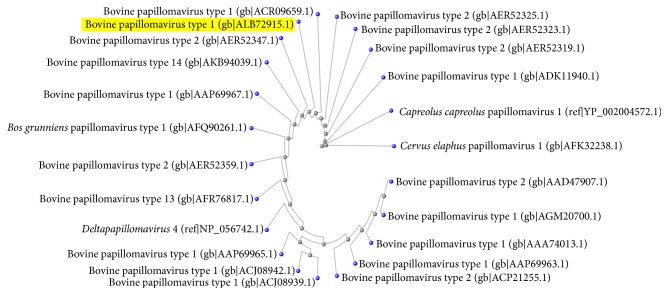
Distance tree created using the NCBI database after searching for Iraqi BPV-1 transforming protein E5. A 100% identity match was found with a BPV-1 E5 protein sequence isolated from a South African sarcoid-affected zebra (accession number ACR09659) and a 98% identity match was found with a BPV-1 E5 protein sequence isolated from an equine sarcoid in the United Kingdom (accession number AAP69967). The tree was created using the neighbor-joining method.

We were able to obtain a complete E5 gene sequence, which we translated into an amino acid sequence that we deposited into GenBank under accession number ALB72915. The amino acid sequence was mpnlwfllfl glvaamqlll llfmllfflv ywdhfecscs nlpf. A BLAST search of the NCBI database using the identified Iraqi BPV-1 transforming protein E5 sequence showed a 100% identity match with a BPV-1 E5 sequence obtained from a South African sarcoid-affected zebra (accession number ACR09659) and a 98% identity match with a BPV-1 E5 sequence obtained from an equine sarcoid in the United Kingdom (accession number AAP69967). A distance tree was created using the NCBI database, and it confirmed these results (Figure 4).

### 3.5. Protein Structure

**Figure 5:**
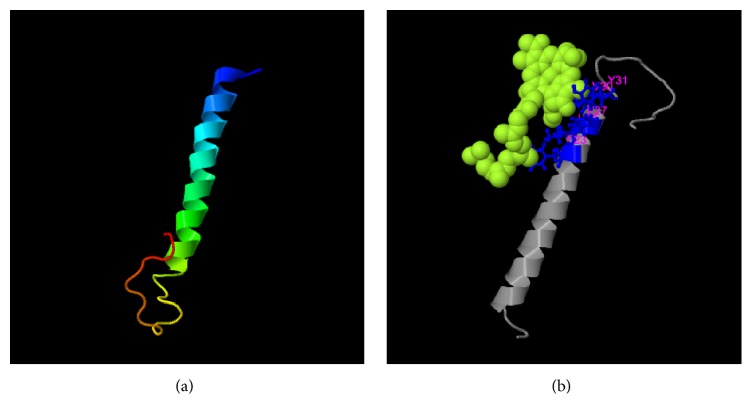
Predicted protein structure for the complete transforming transmembrane protein E5 generated using the I-TASSER server. (a) The predicted E5 protein structure created by the program. (b) A predicted ligand-binding site on E5, this site is a possible location for E5/PDGF*β*-R interactions.

The protein structure of the complete amino acid sequence of transforming transmembrane protein E5 from the isolated Iraqi BPV-1 was analyzed using the I-TASSER server, an Internet-based software program that can be used to generate protein structure and function predictions. I-TASSER generates high-quality predictions of the 3D structures and biological functions of protein molecules based on their amino acid sequences (Figure 5(a)). Based on this analysis, a ligand-binding site was predicted on E5 that facilitates its binding to PDGF*β*-R (Figure 5(b)).

## 4. Discussion

In the current work, clinically and histologically diagnosed cases of bovine papillomatosis were studied to identify and characterize the BPV-1 genotype which was the most prevalent in the central Iraq region, which is a major location for large cattle breeding farms. The identification and characterization of the BPV-1 genotype present in this region are important for effective disease control. We used genotype-specific primers to identify and characterize BPV-1 confirmed by sequencing and phylogenetic analysis, which are considered to be the best methods for these purposes according to the literature [25, 26].

Histological findings showed that the wart specimens evaluated in this study were cutaneous fibropapillomas, which showed characteristic features of papillomatosis as described by Zachary and McGavin [27]. In our molecular analysis, BPV-1 DNA was detected in 121 samples. Amplicons obtained by PCR reactions were submitted for sequencing and found to be identical, confirming the presence of BPV-1, which belongs to the genus* Deltapapillomavirus*. The current study revealed that highly pathogenic BPV-1 is widespread in Iraq; this genotype is associated with the development of cutaneous papillomatosis (fibropapilloma) [28]. To the best of our knowledge, this is the first study to report the presence of BPV-1 in Iraq.

In the present investigation, we were able to obtain a complete E5 gene sequence. Interestingly, based on distance analysis, this sequence was found to exhibit high similarity to E5 amino acid sequences isolated from a South African zebra and from equine sarcoids in the United Kingdom. These results suggest the possibility of disease transmission via British army horses during the 1920s while they were present in Iraq. Another possibility is that BPV-1 in Iraq originated from the importation of livestock from other countries because Holstein Friesian cattle [17, 18] are heavily imported into Iraq. Fibropapilloma viruses encode the most highly conserved E5 proteins [29]. To characterize BPV-1, the structure of the E5 protein and its ligand-binding site were predicted using a special program. E5 is the smallest known oncoprotein that regulates cell transformation. This regulation is achieved via the activation of PDGFR-*β* [30]. The E5 protein forms a dimer in transformed infected cells. The dimer contains a membrane-spanning segment that directly binds to the transmembrane domain of PDGF*β*-R. This binding then induces receptor dimerization, autophosphorylation, and sustained mitogenic signaling [3, 31]. The BPV-1 E5 protein has been shown to be localized to transformed basal keratinocytes within fibropapilloma tissues [32]. Targeting E5 as a possible therapeutic agent has also been described [10]. Thus, studies of this protein are important as well as other virus proteins to better understand the carcinogenesis molecular pathway such as Calpain 3 that is expressed in papillomavirus-associated urothelial cancers of the urinary bladder in cattle [33].

The current study is the first aimed at the detection and characterization of BPV-1 genotype in central Iraq. The results of this study are important for aiding in the development of prophylactic and therapeutic measures to reduce the economic losses associated with bovine papillomatosis in Iraq.

## Figures and Tables

**Table 1:** PCR amplification protocol.

Step	Temperature	Duration	Cycles
Initial denaturation	95°C	3 min	1
Denaturation	95°C	15 sec	35
Annealing	53.5°C	15 sec	
Extension	72°C	15 sec	
Final extension	72°C	1 min/kb	1
